# Symptoms of Autism Spectrum Disorders Measured by the Qualitative Checklist for Autism in Toddlers in a Large Sample of Polish Toddlers

**DOI:** 10.3390/ijerph19053072

**Published:** 2022-03-05

**Authors:** Alicja Niedźwiecka, Ewa Pisula

**Affiliations:** Department of Health and Rehabilitation Psychology, Faculty of Psychology, University of Warsaw, Stawki 5/7, 00-183 Warsaw, Poland; ewa.pisula@psych.uw.edu.pl

**Keywords:** screening for autism spectrum disorders, gender differences, Quantitative Checklist for Autism in Toddlers (Q-CHAT), developmental delay, familial risk for autism spectrum disorders

## Abstract

This study aimed to assess some early symptoms of autism spectrum disorders (ASD) measured by a screening tool developed for an early detection of ASD. We investigated if the early symptoms were associated with toddlers’ age, gender or ASD familial risk status. We used the Polish version of the Quantitative Checklist for Autism in Toddlers (Q-CHAT) to assess 1024 children aged 16 to 36 months. The sample included four groups of participants: typically developing toddlers, toddlers with parent-reported ASD-specific concerns, toddlers at risk for autism due to having an older sibling with ASD, and toddlers with a developmental delay. We found that mean Q-CHAT scores were significantly higher in boys than in girls. We did not find any associations between Q-CHAT scores and age. We observed that toddlers with a familial risk for ASD and those with a developmental delay scored significantly higher than controls. We collated these results with previous studies that used the Q-CHAT and other instruments.

## 1. Introduction

Autism spectrum disorders (ASD) are a group of neurodevelopmental disorders characterized by social communication difficulties and restricted patterns of behavior [1,2]. The prevalence of ASD is estimated at 1–2% of the general population [3,4], although the epidemiological data vary across countries [5]. The etiology of ASD remains unknown. The available data suggest a complex interplay of a variety of genetic, neurobiological, and environmental factors [6,7]. As ASD biomarkers are yet to be established, diagnosis is still made on the basis of behavioral symptoms. Behavioral symptoms of ASD may be present in various groups of children, not only in those meeting the diagnostic criteria for ASD, e.g., typically developing children [8] and children with developmental delay/intellectual disability [9,10]. The issue still requires a better understanding in order to support the development of young children at risk of ASD more effectively.

Since supporting early development can bring significant effects and improve life prospects of children with ASD [11,12], measures are taken to identify symptoms as early as possible in order to initiate appropriate intervention. Screening tools with good psychometric properties are needed to increase the chances of recognizing ASD early and providing children with professional support. One of the tools useful for ASD screening is the Quantitative Checklist for Autism in Toddlers (Q-CHAT; 13).

### 1.1. Screening for ASD with the Q-CHAT

The Q-CHAT is a screening instrument for ASD for toddlers aged 18 to 24 months. This 25-item questionnaire is completed by caregivers. Previous studies with British [13,14], Italian [15,16], and Singaporean [17] participants demonstrated acceptable internal consistency, test-retest stability, and validity. Furthermore, a prospective study demonstrated the usefulness of the English version of the Q-CHAT as a screening tool for toddlers aged 18–30 months [18]. A 10-item, “red flags” version of the Q-CHAT (the Q-CHAT-10) has also been developed [19]. With good reliability, accuracy and high specificity and sensitivity, the authors suggest that this tool could be used by frontline medical professionals to identify children who need further assessment.

There are several other instruments designed for early screening for symptoms of ASD among toddlers, including the Modified Checklist for Autism in Toddlers (M-CHAT) [20] and a newer version, the Modified Checklist for Autism in Toddlers, revised with follow up (M-CHAT-R/F) (Robins et al., 2014) [21], the Social Attention and Communication Surveillance [22] and a revised version of the instrument [23] and the Pervasive Developmental Disorder Screening Test–II [24]. The Q-CHAT and the M-CHAT and the M-CHAT-R/F are available in Polish, however, these versions have not yet been validated, and their psychometric properties are unknown. They are free of charge and easily available instruments. The 4-point rating scale of the Q-CHAT may be more user-friendly, as it does not force respondents to choose between yes and no answers, as the M-CHAT does.

Several papers presented studies in which translated versions of the Q-CHAT were used: Spanish [25], Persian [26], and Korean [27]. Translations into other languages also exist [28]. However, information about the tool’s validity remains limited, especially with respect to measuring early symptoms of ASD in different groups of children at risk for ASD and in typically developing children. Information on the usefulness of Q-CHAT in measuring the severity of ASD symptoms in various groups of toddlers may provide knowledge valuable for clinical practice. It is also essential to examine the relations between the age and gender of children and the autistic symptoms assessed with the Q-CHAT.

### 1.2. Factors Related to the Diagnosis of ASD in Early Development

Age  Symptoms of ASD emerge over the first 18 months of a child’s life [29], with some symptoms appearing already in the first year of life [30]. The severity of ASD symptoms increases with age [31]. However, a question remains whether these symptoms are related to children’s age in toddlers in the general population. Magiati et al. [17] reported that in their prospective study, Q-CHAT scores were significantly lower at the age of 24 months than at the age of 18 months. It is conceivable that, in the general population, the incidence of some autistic-like behaviors decreases as children’s development progresses.

Gender  Males are four times more likely than females to be diagnosed with autism [32]. In a Swedish population study, screening for ASD among toddlers revealed a greater prevalence in boys than in girls [33]. Furthermore, early in the development, some sub-clinical symptoms seem to be more prominent in males than in females in the general population [34]. Unfortunately, the effect gender in large screened cohorts of toddlers and young children is not always reported e.g., ref. [35].

Some studies have found clear differences in developmental profiles of boys and girls with ASD early in life [36,37]. However, the results of other studies did not support the existence of differences between preschool boys and girls with suspected autism [38] or found a pattern of overall similarities with few gender differences [39]. Several studies that used the Q-CHAT reported gender differences with boys scoring higher than girls [14,40,41], while some other studies did not demonstrate such differences [27]. The latter may suggest that girls and boys are less dissimilar than previously thought, but an alternative interpretation could be that currently available instruments under-identify difficulties among girls [38]. More studies are needed to improve our understanding of gender differences in ASD among toddlers and young children [42].

Parents’ first concerns  Early diagnosis of ADS relies to a great extent on symptoms reported by parents to health care providers. Studies have shown that parents of children later diagnosed with ASD notice first symptoms earlier than parents of children with developmental delay or other developmental atypicalities [43,44]. The earliest age when parents’ first concerns are predictive of ASD outcomes may be 12 months and is specific to younger siblings of children with a diagnosis of ASD [45]. Early in the second year of life, parental concerns may include behaviors that are not specific to ASD (motor atypicalities, passivity, emotional functioning, hyperactivity, and sleep problems) [46]. In the second and third year of life, the area that raises particular concerns is communication [47,48,49]. According to a study by Vostanis and colleagues [50], at 12 to 18 months of age, play behaviors and a lack of referential gestures in-between were the best predictors of a later ASD diagnosis. Of particular interest for this paper, key areas of development that are relevant to a later diagnosis of ASD, i.e., communication and play, are addressed by the Q-CHAT.

Familial risk  Younger siblings of children diagnosed with ASD are several times more likely to be diagnosed with ASD than children in the general population [51,52]. Some subclinical symptoms, described as the broader autism phenotype, can also be observed in this group see a review in: [53]. The severity of probands’ symptoms in the area of language and communication is associated with developmental outcomes in the same area in their younger siblings [54]. Certain early behaviors differentiate between toddlers with a familial risk of ASD who go on to receive a diagnosis of ASD and those who develop typically [55]. This has also been demonstrated in a study in that used the short version of the Q-CHAT, the Q-CHAT-10 [56]. It is important to devise and perfect effective tools for an early detection of ASD in this group of children.

Developmental delay  ASD and developmental delay are highly comorbid [57,58,59]. An early differentiation between children who need an intervention targeted specifically at symptoms of ASD and those who have a developmental delay without ASD is crucial for obtaining the best therapeutic outcomes possible see [60]. However, early screening tools are sensitive to developmental atypicalities, but they may have relatively low specificity e.g., [61]. It may be difficult to distinguish between ASD and other developmental disabilities early in a child’s life because they share certain symptoms, such as atypicalities in motor development [62], motor stereotypies [63], and language delay [64]. However, it has been demonstrated that the Q-CHAT does differentiate children with ASD from those with developmental delays [16].

### 1.3. Current Study

We used a Polish version of the Q-CHAT to examine the symptoms of ASD in a sample of Polish toddlers, aged 16 to 36 months, without a formal diagnosis of ASD, any genetic syndromes, or brain abnormalities. In Poland, the proportion of children with ASD who receive a formal diagnosis at this age is very small. It is worth examining Q-CHAT in typically developing children, children with some ASD-specific concerns reported by parents, who were at the time of assessment at an initial stage of a diagnostic process in diagnostic and intervention centers, younger siblings of children with ASD and toddlers diagnosed with a developmental delay. Before conducting between-group comparisons, we examined the effects of age and gender on Q-CHAT scores. We expected to find a significant negative correlation between Q-CHAT scores and age and we predicted that boys would score higher than girls. We then compared Q-CHAT scores in four subgroups of participants: typically developing toddlers (the TD group), toddlers with parent-reported ASD-specific concerns (the ASD-concerns group), toddlers at risk for autism due to having an older sibling with ASD (the ASD-sibling group), and toddlers with a developmental delay (the DD group). All the children whose parents indicated that they had a sibling with ASD have been assigned to the ASD-sibling group, including those whose parents reported ASD-specific concerns. We predicted that children with ASD concerns reported by parents and those with a familial risk for ASD would score higher than controls. We also expected children with a developmental delay to score higher than controls.

## 2. Materials and Methods

### 2.1. Study Design and Procedures

Questionnaires were completed by the primary caregiver (84% mothers) as part of several studies at home or in day care, early intervention centers and clinics across Poland. Participants were recruited by professionals providing day care or early diagnosis and intervention, through flyers and posters in healthcare centers and nurseries, and media ads. All parents provided written informed consent. The studies were approved by the local institution’s ethics committee and conformed to the Declaration of Helsinki.

### 2.2. Participants

Altogether, 1201 participants took part. Eighty-eight participants were excluded because of missing data in Q-CHAT forms. Forty participants were excluded because they were younger than 16 months and therefore ineligible for the study. Next, we removed participants with missing data for key demographic variables: child’s age (*n* = 3), child’s gender (*n* = 1) or information regarding child’s development (whether the child was developing typically, had a developmental delay or if his/her parents had any ASD-specific concerns) (*n* = 18). Finally, we removed participants with hearing impairments (*n* = 3), epilepsy, brain anomalies, or genetic disorders (e.g., Down syndrome, Williams syndrome; *n* = 16), and clinically confirmed ASD (*n* = 8; *M* Q-CHAT score = 41, min = 23, max = 53). The ASD group was excluded as it was too small for between-group comparisons. The final sample included 1024 participants (the TD group, *n* = 585; the ASD-concerns group, *n* = 252; the ASD-sibling group, *n* = 67; and the DD group, *n* = 120).

Table 1 presents the characteristics of the sample. The mean age of participants was 24.96 months (*SD* = 5.09, min = 16, max = 36). Boys (*n* = 603) outnumbered girls (*n* = 421), X^2^ (1, 1024) = 32.348, *p* < 0.001. Ten percent of the children were born preterm (<37 weeks gestational age at birth) (missing data *n* = 142). Forty-two percent had one or more siblings. 73% of mothers had higher education (missing data *n* = 148) and 57% of fathers had higher education (missing data *n* = 373). Participants lived all across Poland, in towns and villages of varying populations.

Regarding the characteristics of the four subgroups in our study, there was a significant but weak main effect of age, *F* = [3, 1020] = 17.940, *p* < 0.001, η_p_^2^ = 0.050. Toddlers in the ASD-sibling group were significantly younger than those in the TD group, *t* = −3.061, *p* < 0.001, BCa 95% CI [−4.991, −1.130]. They were also significantly younger than toddlers in the ASD-concerns group, *t* = −2.574, *p* = 0.003, BCa 95% CI [−4.522, −0.626], and toddlers in the DD group, *t* = −4.172, *p* < 0.001, BCa 95% CI [−6.333, −2.011]. There was no significant age difference between the TD and the DD group, *p*s > 0.05. Toddlers in the ASD-concerns group were significantly younger than those in the TD group, *t* = −1.566, *p* < 0.001, BCa 95% CI [−2.301, −0.830]. Finally, toddlers in the ASD-concerns group were significantly younger than those in the DD group, *t* = −1.598, *p* = 0.044, BCa 95% CI [−3.170, −0.027]. Therefore, age was entered as a covariate in the comparisons of Q-CHAT scores.

In the TD group, there was no significant difference in the number of boys and girls, *p* > 0.05. In the other three groups, boys outnumbered girls (ASD-concerns: X^2^ (1, 252) = 55.254, *p* < 0.001; ASD-sibling: X^2^ (1, 67) = 14.343, *p* < 0.001; DD: X^2^ (1, 120) = 7.500, *p* = 0.006).

### 2.3. The Instrument

The original version of the Q-CHAT was translated into Polish by a psychologist and a psychiatrist specialized in ASD. This translation has been verified by a professional translator, then the Polish version was blindly translated back into English by a different translator. The original English and the back-translated versions were then compared by a native English speaker and necessary edits were made see [65]. Corrections concerned only minor phrases that did not significantly alter the meaning of the items. As in the original version of the Q-CHAT, participants scored each of the 25 items on a scale from 0 to 4, with higher scores indicating atypical behavior symptomatic of ASD. The Q-CHAT forms in different languages can be found of the website of the Autism Research Centre: https://www.autismresearchcentre.com/tests/quantitative-checklist-for-autism-in-toddlers-q-chat/, (accessed on 3 January 2022).

The internal consistency of the instrument in this study was examined using the Cronbach’s method. The alpha coefficient obtained for the Q-CHAT scores was 0.784 for the entire sample. For the TD group, the alpha coefficient was 0.779, for the ASD-concerns group 0.767, for the ASD-sibling group 0.771, and for the DD group 0.673.

Additionally, a demographic questionnaire was used to collect some basic socio-demographic data about participants, including the children’s age, gender, gestational age at birth, number of siblings, ASD diagnosis in close relatives, developmental concerns and health issues, parents’ education level, and the family’s place of residence. Some of the data was only available for subgroups of participants.

### 2.4. Analyses

We began our analyses by testing the normality of the distribution of Q-CHAT scores in our sample.

We used the Pearson’s correlation to determine whether Q-CHAT scores were related to participants’ age in the entire sample. Next, we conducted a univariate ANOVA with the age as a covariate to compare Q-CHAT scores of boys and girls and an independent-samples *t*-test to compare boys’ and girls’ scores in specific items. Finally, we used a univariate ANOVA with the age as a covariate to compare the scores of four groups of participants: the TD group, the ASD-concerns group, the ASD-sibling group, and the DD group.

## 3. Results

### 3.1. Distribution of Q-CHAT Scores

**Figure 1:**
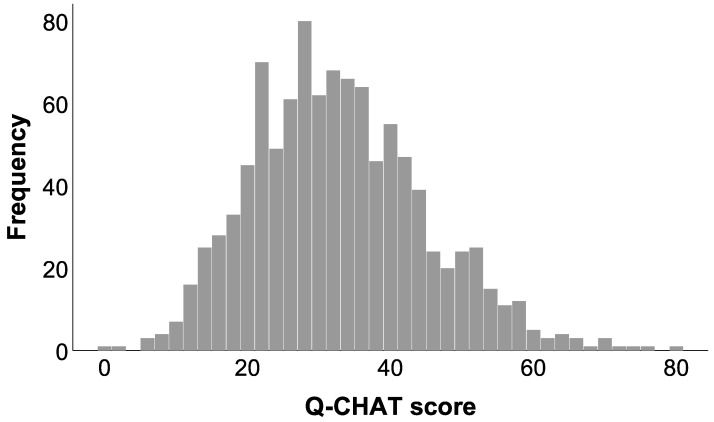
The distribution of Q-CHAT scores.

Figure 1 presents the distribution of Q-CHAT scores for the entire sample (*M* = 32.285, *SD* = 12.157). The distribution significantly differs from the normal distribution, *D* = 0.054, *p* < 0.001. With a skewness of 0.492 (SE = 0.076) and a kurtosis of 0.284 (SE = 0.153), the distribution has a positive skew and is pointy.

### 3.2. Age and Q-CHAT Scores

We ran zero-order correlations to determine whether Q-CHAT scores were associated with the age of participants. The correlation was non-significant, *r* = −0.040, *p* = 0.202.

### 3.3. Gender and Q-CHAT Scores

A univariate ANOVA with age as covariate revealed a significant difference in Q-CHAT scores between boys and girls, *F* = [1, 121] = 7.063, *p* = 0.008, η_p_^2^ = 0.007. The main effect of age was non-significant, *p* > 0.05. Boys (*M* = 33.149, *SD* = 12.114) scored significantly higher than girls (*M* = 31.048, *SD* = 12.157). The effect size was, however, very small.

An item-by-item analysis revealed that boys scored significantly higher than girls (indicating worse performance) on the following items: 1. Look when call name, 2. Eye contact, 4. Understand child’s speech, 5. Protoimperative pointing, 6. Protodeclarative pointing, 8. Number of words, 9. Pretend play, 10. Follow a look, 15. Offer comfort, 19. Gestures. Girls scored significantly higher than boys on two items: 22. Maintenance of interest, and 24. Oversensitive to noise (see Table 2).

### 3.4. Parental Concerns, ASD Familial Risk, Developmental Delay, and Q-CHAT Scores

We used the GLM to determine whether there were any significant differences in Q-CHAT scores between four groups of participants: the TD group, the ASD-concerns group, the ASD-sibling group, and the DD group. Age was entered as a covariate.

**Figure 2:**
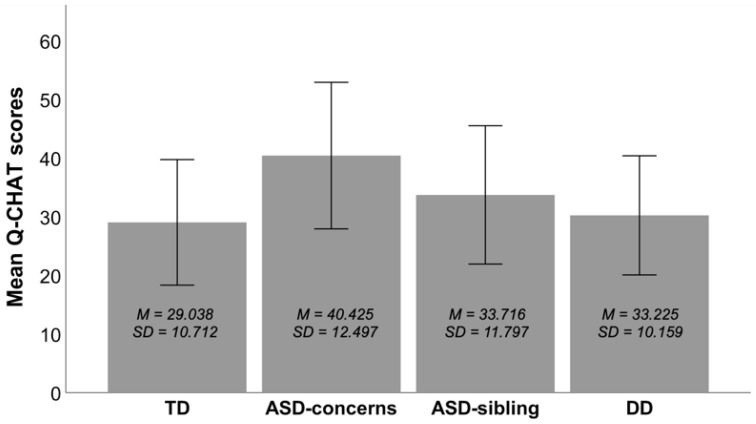
Mean Q-CHAT scores by group. Error bars represent standard deviation of mean. Note: TD, typical development; ASD, autism spectrum disorders; DD, developmental delay.

The analyses revealed a significant main effect of group, *F* = [1, 1019] = 62.056, *p* < 0.001, η_p_^2^ = 0.154 (see Figure 2). The main effect of age was non-significant, *p* > 0.05. Planned comparisons showed that children from the ASD-concerns group scored significantly higher than the TD group, *t* = 11.455, *p* < 0.001, BCa 95% CI [9.786, 13.123]. Furthermore, children from in the ASD-sibling group scored significantly higher than the TD group, *t* = 4.858, *p* = 0.001, BCa 95% CI [1.968, 7.747]. There was no significant difference between the TD and DD groups, *p* > 0.05.

Bonferroni-corrected pairwise (*post-hoc*) comparisons revealed that children from the ASD-concerns group scored significantly higher than those with a familial risk for ASD, *t* = 6.783, *p* < 0.001, BCa 95% CI [2.726, 10.761], and children with a developmental delay, *t* = 10.178, *p* < 0.001, BCa 95% CI [6.947, 13.408]. There was no significant difference between children with familial risk for ASD and those with developmental delay, *p* > 0.05.

## 4. Discussion

In this study, we assessed the symptoms of autism spectrum disorders with the Quantitative Checklist for Autism in Toddlers in a large sample of Polish toddlers aged 16–36 months. We examined the associations between participants’ age and Q-CHAT scores and compared the scores of boys and girls. We also compared the scores among four groups of participants: toddlers with parent-reported ASD-specific concerns, toddlers having an older sibling with ASD, toddlers with a developmental delay and typically developing controls. Toddlers with parent-reported ASD-specific concerns who were undergoing a diagnosis for ASD obtained the highest mean Q-CHAT scores. Furthermore, the mean score in this group (*M* = 40) was higher than the cut-off point of 39 proposed by Allison and colleagues [18]. Although cut-off points cannot be generalized from one language version to another, we can tentatively assume that the Polish Q-CHAT distinguishes toddlers with ASD-specific concerns from controls and from toddlers with developmental delay.

### 4.1. Age, Gender, and Q-CHAT Scores

Q-CHAT scores were not significantly associated with participant’s age in our study. This result is inconsistent with the findings reported by [17], who demonstrated in a longitudinal study that Q-CHAT scores decreased from 18 to 24 months. A longitudinal design possibly allowed the authors to detect age-related changes that were not observed in our research.

Allison et al. [13], Aueyung et al. [14], and Ruta et al. [15] all reported significant gender differences in Q-CHAT scores, with boys scoring higher than girls. Another study, in which a different screening tool was used, the M-CHAT) [20], also found that 18-month-old boys in the general population scored higher than girls [34]. Our data is consistent with those results. An item-by-item analysis revealed that boys showed more elevated symptoms, especially in the social communication domain. This is consistent with a previous study with toddlers and young children that found more elevated social communication symptoms in girls than in boys [66]. Similarly, Little, Wallisch, Salley, & Jamison [67], who studied early parental concerns regarding their child’s development, found that parents of boys diagnosed later with ASD reported more concerns regarding social interaction compared with girls with ASD or children with other developmental disabilities.

Altogether, these results suggest that a careful examination of the Q-CHAT, including item analysis, may be necessary to better understand gender effects. Perhaps different cut-off points should be established for boys and girls for screening tools such as the Q-CHAT, as is the case for the Macarthur-Bates Communicative Development Inventories [68,69]. The issue is the subject of lively discussion among researchers cf. e.g., [70], and a commentary by Ratto, ref. [71]. Efforts are also being made to develop screening instruments that are more sensitive to the female phenotype in ASD. Some examples are an extension of the Autism Spectrum Screening Questionnaire for school-aged children and youth, which includes additional items that capture some of the symptoms that are more specific to girls [72], and the Girls Questionnaire for Autism Spectrum Condition (GQ-ASC) [73], used to measure autism spectrum symptoms in adult women.

### 4.2. ASD Familial Risk, Parental Concerns, and Q-CHAT Scores

Studies with younger siblings of children with ASD enrich our understanding of the disorders and their early symptoms [74,75]. Younger siblings of children with ASD in our study scored lower than children with parent-reported ASD-specific concerns, but higher than typically developing controls. We had anticipated this result, as some of these children may go on to receive a diagnosis of ASD. Furthermore, some developmental atypicalities can also be observed in those younger siblings of children with ASD who do not go on to receive a diagnosis [76]. The scores of children in the ASD-sibling group did not differ significantly from the scores of children with a developmental delay. This result is consistent with the one presented by Raza et al. [56], who reported low specificity of the Q-CHAT-10 in a Canadian sample of participants at high familial risk for ASD. Altogether, these results suggest that the Q-CHAT may have good sensitivity but relatively low specificity. Notably, the DD group in our study was heterogenous and the children had not yet undergone any ASD-specific assessment. Some of those children may go on to receive a diagnosis of ASD, just as some of the children in the ASD-sibling group. Thus, longitudinal studies are needed to further examine the specificity of the Q-CHAT.

### 4.3. The Polish Q-CHAT and the Other Versions of the Instrument

Our study provides some preliminary information regarding the psychometric properties of the Polish Q-CHAT. Unlike the distributions of Q-CHAT scores presented by Allison et al. [13], Magiati et al. [17], and Ruta et al. [15], the distribution in our sample differed from the normal distribution. However, its shape was close to normal. This discrepancy can be accounted for by some differences in the characteristics of the samples. Specifically, in the study by Allison et al. [13], the sample included 779 typically developing children and 160 children with ASD. In the studies by Auyeung et al. [14], Magiati et al. [17], and Ruta et al. [15], only typically developing toddlers were tested. On the other hand, in the study by Mohammadian et al. [26], half of the sample had a diagnosis of ASD.

Despite recent efforts, relatively little is known about the usefulness of the Q-CHAT in the early identification of ASD symptoms in non-English-speaking populations. This is unfortunate, as parental evaluations of early ASD symptoms, including socio-communication problems, may be related to social and cultural conditions [77]. Parents in different cultures value different social skills, therefore they may ascribe different importance to impairments in this domain [78]. Several studies demonstrated cultural differences in parental reports of ASD symptoms [79,80,81,82,83]. Although cultural differences are apparent, Al Maskari, Melville, and Willis [84] note that modifications beyond a simple translation are rarely made in screening tools. While the psychometric properties of translated versions vary, little attention is given to cultural adaptations [85].

### 4.4. Limitations

This study is limited by the characteristic of the sample. First, the sample is not random. A portion of the data was collected in early intervention centers. Consequently, participants with parent-reported developmental concerns and those with a developmental delay were overrepresented. This also led to an overrepresentation of boys. Second, there were some significant age differences among the four subgroups in the study. Finally, participants in the ASD-concerns group did not have a diagnosis of ASD at the time of assessment with the Q-CHAT, therefore we were unable to analyze the instrument’s sensitivity and specificity or to establish cut-off points. More research is needed in order to adapt the Q-CHAT for use in Poland.

## 5. Conclusions

This study of a large sample of Polish toddlers demonstrated that the Q-CHAT, an early screening tool for ASD symptoms, differentiated between children at familiar risk for ASD and controls. It also differentiated between children with developmental delay and controls. As in certain other studies, gender differences were found with boys scoring higher than girls, indicating higher levels of symptoms of ASD in boys. Future studies ought to address the issue of cultural adaptations of the Q-CHAT as there could be cultural variation in the perception of symptoms of ASD. Finally, results regarding early gender differences in symptoms of ASD are inconsistent; therefore, the question of gender effects requires attention.

## Figures and Tables

**Table 1:** The characteristics of the sample.

	TD	ASD-Concerns	ASD-Sibling	DD
*n* Boys/Girls	294/291	185/67	49/18	75/45
Mean age in months (SD)	25.617 (4.512)	24.052 (5.704)	21.478 (4.894)	25.650 (5.480)

Note: TD, typical development; ASD, autism spectrum disorders; DD, developmental delay.

**Table 2:** Q-CHAT item scores of boys and girls: descriptives and test statistics.

Item	*M* (*SD*) Boys	*M* (*SD*) Girls	Test Statistic
1. Look when call name	1.170 (0.896)	0.957 (0.875)	1.995 *
2. Eye contact	0.907 (0.828)	0.786 (0.806)	2.336 *
3. Line objects up	1.880 (1.385)	1.922 (1.302)	−0.497
4. Understand child’s speech	1.781 (1.438)	1.469 (1.338)	3.557 *
5. Protoimperative pointing	1.176 (1.325)	0.950 (1.187)	2.855 *
6. Protodeclarative pointing	1.512 (1.422)	1.143 (1.236)	4.428 *
7. Interest maintained by spinning object	1.221(1.101)	1.112 (1.134)	1.531
8. Number of words	1.652 (1.215)	1.397 (1.098)	3.499 *
9. Pretend play	1.643 (1.294)	1.337 (1.219)	3.846 *
10. Follow a look	1.262 (1.251)	1.031 (1.107)	3.115 *
11. Sniff/lick unusual objects	1.181 (1.199)	1.107 (1.209)	0.967
12. Use of hand as tool	1.624 (1.273)	1.639 (1.307)	−0.188
13. Walk on tiptoes	1.269 (1.073)	1.323 (1.051)	−0.805
14. Adapt to change in routine	0.924 (0.904)	0.641 (0.904)	−0.294
15. Offer comfort	2.027 (1.303)	1.805 (1.282)	2.693 *
16. Do same thing over and over again	1.665 (1.359)	1.662 (1.359)	0.036
17. Typicality of first words	1.449(1.441)	1.323 (1.406)	1.395
18. Echolalia	1.443 (1.296)	1.580 (1.315)	−1.652
19. Gestures	1.309 (1.342)	1.078 (1.256)	2.804 *
20. Unusual finger movements	0.982 (1.337)	0.988 (1.279)	0.076
21. Check reaction	1.068 (1.109)	1.186 (1.214)	−1.580
22. Maintenance of interest	1.126 (1.023)	1.261 (1.034)	−2.072 *
23. Twiddle objects repetitively	1.111 (1.269)	1.100 (1.225)	0.143
24. Oversensitive to noise	1.080 (1.023)	1.221 (1.045)	−2.156 *
25. Stare at nothing with no purpose	0.796 (1.075)	0.741 (1.047)	0.813

* *p* < 0.05.

## Data Availability

Data are available from authors upon request.
